# Immunotherapy in transplanted patients: A special population that can no longer be ignored

**DOI:** 10.1111/dth.14975

**Published:** 2021-05-26

**Authors:** Marco Rubatto, Martina Merli, Andrea Agostini, Gianluca Avallone, Luca Mastorino, Paolo Fava, Luigi Biancone, Maura Rossetti, Maria Teresa Fierro, Simone Ribero, Pietro Quaglino

**Affiliations:** ^1^ Department of Medical Sciences, Dermatologic Clinic University of Turin Medical School Torino Italy; ^2^ Department of Medical Sciences, Nephrology, Dialysis and Transplantation U University of Turin Medical School Turin Italy

Dear Editor,

Checkpoint inhibitors, such as anti‐programmed death‐1 (anti‐PD‐1) and programmed death‐ligand 1 (anti‐PD‐L1), have revolutionized treatments for advanced skin diseases. In 2018, cemiplimab was approved from the US Food and Drug Administration for the treatment of advanced or metastatic cutaneous squamous cell carcinoma (cSCC) not eligible for surgical treatment or curative radiotherapy.^1^

Cemiplimab is a monoclonal antibody that works by binding to PD‐1 and blocking its interaction with PD‐L1. This blockage enhances antitumor activity and upregulates cytotoxic T cells.

During the American Society of Clinical Oncology 2020 Congress, the results of the EMPOWER‐CSCC, a phase 2 study that evaluates cemiplimab in monotherapy for patients with metastatic or unresectable locally advanced cSCC, evidence a response rate of 46.1% with 16.1% complete responses.^2^

Studies regarding immunotherapy usually excluded patients with autoimmune diseases as history of solid organ transplantation.

On the contrary, the French and American studies confirmed the response data obtained in clinical trials also in real life. Twenty‐six percent of French study patients were immunocompromised while the American one included patients with relevant comorbidity that are commonplace in real life.^3^, ^4^

Given the proven effectiveness of immunotherapy, there is often a dilemma about the use of immunotherapy drugs in these special populations that have a higher risk of developing cSCC from 65 to 250 compared to the general population.^5^

We report a case of a 55‐year‐old woman who came to our observation, suffering from Alport syndrome with three previous renal transplants performed. Following the first renal transplant, the patient was treated with antimetabolites of DNA synthesis (azathioprine and mycophenolate mofetil) and then with mTOR inhibitors (tacrolimus and everolimus). In recent years, the use of mechanistic target of rapamycin (mTOR) inhibitors (a class of molecules that inhibit the mammalian target of rapamycin) has demonstrated its effectiveness in decreasing the risk of developing cSCC in organ transplant recipients. Their use is currently recommended in transplanted patients who had already developed skin cancers.^6^

In spring 2020, cSCC was diagnosed on her left wrist and the exeresis was postponed due to the COVID emergency, with no oncological radicality. Over the following 30 days, the disease spread to arm and axilla, involving lymph nodes (Figure 1).

**FIGURE 1:**
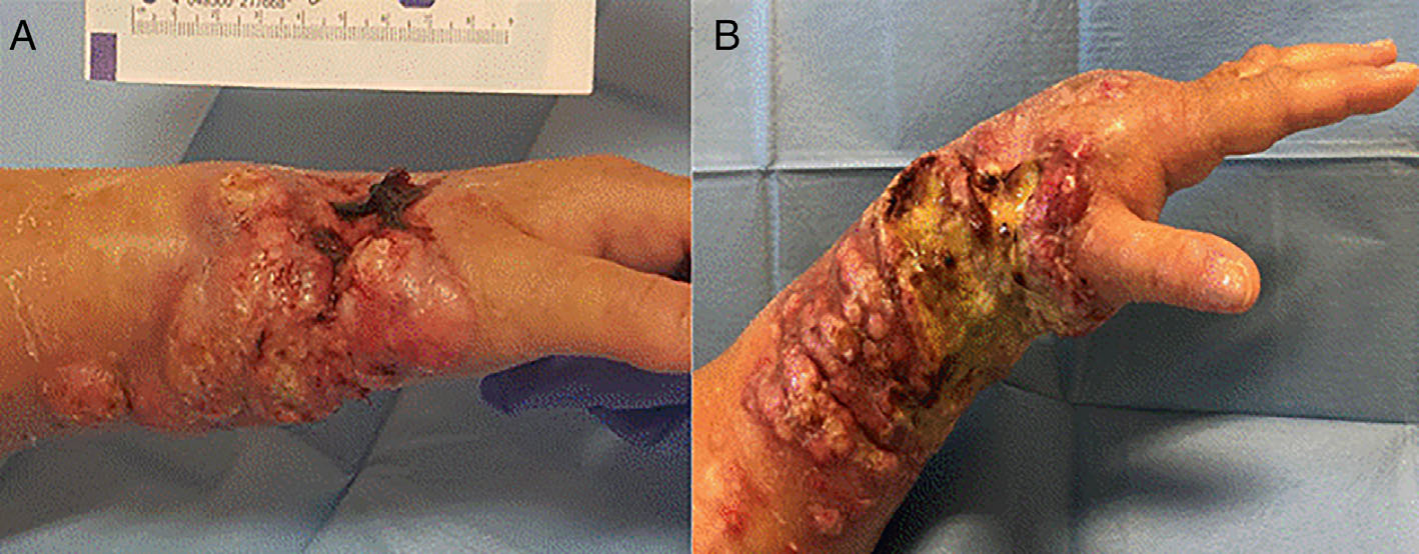
A, Cutaneous squamous cell carcinoma (cSCC) on the left arm before starting the therapy with cemiplimab. B, cSCC on the left arm after the third infusion of cemiplimab

In September, the patient began treatment with cemiplimab and after four administrations no adverse events were observed and the renal function has been maintained. However, clinically, the arm appears edematous with necrotic ulcers compromising its function, compatible with a progression of soft tissue disease.

The computerized tomography (CT) scan after 3 months of therapy, compared with the previous CT scan, showed a volumetric increase of the known adenopathy in the left axillary region.

Despite the aggressiveness of the disease, the clinical response could also be late, so the patient continued the treatment. We will re‐evaluate the patient together with radiotherapists for local treatment and nephrologists for the immunosuppressive therapy.

The largest collection of patients undergoing organ transplantation and immunotherapy treatments in the literature includes only 39 patients. Sixteen of them had organ rejection after an average of 21 days, 11 underwent renal transplantation and 10 needed dialysis.^7^

Currently, only two studies are evaluating immunotherapy in renal transplant recipients.^8^, ^9^

This case showed the importance of a clinical multidisciplinary approach and risk communication to the patient in the management of this special population. The lack of knowledge about the safety and efficacy of immunotherapy in patients with previous transplantation is a major problem. Currently, there are no clear recommendations about these cases. Multicenter studies and common guidelines are needed to better understand the interactions between alloantigens and tumor antigens to establish a therapeutic pathway that maximizes the effects of immunotherapy and preserves the transplanted organ.

The patient has signed written informed consent.

## CONFLICT OF INTEREST

The authors declare no potential conflict of interest.

## AUTHOR CONTRIBUTIONS

Marco Rubatto conceived and designed the manuscript. Andrea Agostini wrote the paper. Gianluca Avallone, Martina Merli, Luca Mastorino and Paolo Fava reviewed the literature and took the pictures. Luigi Biancone, Maura Rossetti, Maria Teresa Fierro, Simone Ribero and Pietro Quaglino reviewed the manuscript and provided comments. All authors gave final approval for publication.

## Data Availability

Data sharing is not applicable to this article as no new data were created or analyzed in this study.
